# Supplementary material for: A high-resolution mRNA expression time course of embryonic development in zebrafish
Source: eLife. 2017 Nov 16;6:e30860. doi: 10.7554/eLife.30860 (PMC5690287; doi:10.7554/eLife.30860)
Supplement: Supplementary file 6. [file elife-30860-supp6.zip › biolayout-clusters-files/Cluster164-genes.html]

Cluster164


# Cluster164: Genes

| Ensembl ID | Gene Name | Chr | Start | End | Biotype |
| --- | --- | --- | --- | --- | --- |
| ENSDARG00000043856 | amd1 | 20 | 2937165 | 2966128 | protein\_coding |
| ENSDARG00000056525 | bcar1 | 18 | 22617193 | 22699007 | protein\_coding |
| ENSDARG00000062801 | erfl3 | 16 | 11138860 | 11251895 | protein\_coding |
| ENSDARG00000032535 | praf2 | 8 | 44899722 | 44910817 | protein\_coding |
| ENSDARG00000020261 | rab2a | 2 | 22163621 | 22189873 | protein\_coding |
| ENSDARG00000058302 | sh3bgrl | 14 | 9099085 | 9114897 | protein\_coding |
| ENSDARG00000091932 | si:ch73-25f10.6 | 19 | 27870004 | 27878559 | protein\_coding |
| ENSDARG00000019060 | snx12 | 21 | 30434137 | 30450344 | protein\_coding |
